# Papillon- Lefèvre Syndrome: Report of a case and its management

**DOI:** 10.4317/jced.50594

**Published:** 2012-02-01

**Authors:** Shabina Sachdeva, Namita Kalra, Pranav Kapoor

**Affiliations:** 1 MDS. Formerly: Senior Resident, Department of Dentistry, University College of Medical Sciences, New Delhi -110095. Presently: Assistant Professor, Faculty of Dentistry, Jamia Millia Islamia, New Delhi -110025; 2 MDS. Professor & Head, Department of Dentistry, University College of Medical Sciences, New Delhi-110095; 3 MDS. Formerly: Lecturer, Department of Dentistry, University College of Medical Sciences, New Delhi -110095. Presently: Assistant Professor, Faculty of Dentistry, Jamia Millia Islamia, New Delhi -110025

## Abstract

Papillon-Lefèvre Syndrome (PLS) is a rare autosomal recessive disorder first described by two French physicians, Papillon and Lefèvre in 1924. The disorder is characterized by diffuse palmoplantar keratoderma and precocious aggressively progressing periodontitis, leading to the premature loss of deciduous and permanent teeth at a very young age. The cutaneous lesions are usually manifested simultaneously with the intra-oral presentations and include keratotic plaques on the palms and soles varying from mild psoriasiform scaly skin to overt hyperkeratosis. The etiopathogenesis of the syndrome is relatively obscure and immunologic, genetic or possible bacterial etiologies have been proposed. Due to the vast degree of periodontal breakdown involved at such an early age, the dental surgeon is often the first to diagnose the syndrome. This paper presents a clinical presentation a 15 year old male diagnosed with Papillon- Lefèvre Syndrome.

** Key words:**Papillon-Lefèvre Syndrome, palmoplantar keratoderma, rapidly progressing periodontitis.

## Introduction

Papillon-Lefèvre Syndrome (PLS) or keratosis palmoplantaris with periodontopathia is a rare autosomal reces-sive disorder characterized by diffuse transgradient hyperkeratosis of the palms and soles and severely destructive, rapidly progressive periodontal disease (1). Associated features may include intracranial calcifications, susceptibility to bacterial infections and mental retardation (2,3).

The disorder is first seen in children in the age group of 1-4 years. No racial or sexual predilection is reported. A genetic predisposition however exists with greater frequency of occurrence in the consanguineous offspring (1,2).

Patients with Papillon-Lefèvre Syndrome often present with severe gingival inflammation and periodontal destruction soon after the eruption of primary teeth, leading to premature loss of the deciduous dentition. Once the primary dentition exfoliates, the gingiva seems to regain its normal appearance. The eruption of permanent teeth, however, re-triggers the aggressive periodontitis, which is unresponsive to conventional periodontal therapy and results in partial or complete edentulism at a very young age.

The cutaneous lesions are usually manifested simultaneously with the intraoral presentations and present as sharply demarcated erythematous keratotic plaques on the palms and soles, which tend to spread onto the dorsal surfaces.

This paper presents a brief overview of Papillon-Lefèvre syndrome and describes the clinical presentations in a case with typical dental and dermatological findings.

## Case Report

A 15 year old male reported to the Department of Dentistry, University College of Medical Sciences, with the chief complaint of having lost most of his teeth and inability to chew with the remaining ‘loose’ teeth.

Medical history revealed that the patient had been suffering from recurrent skin infections since an early age with thickening and subsequent peeling of the skin of his hands and feet, for which he had been undergoing intermittent treatment. His grandfather had similar dermatological lesions.

Past dental history revealed that the patient had lost all his deciduous teeth by about 3 years of age. A number of his permanent teeth also became mobile soon after eruption and were subsequently extracted.

**Figure 1:**
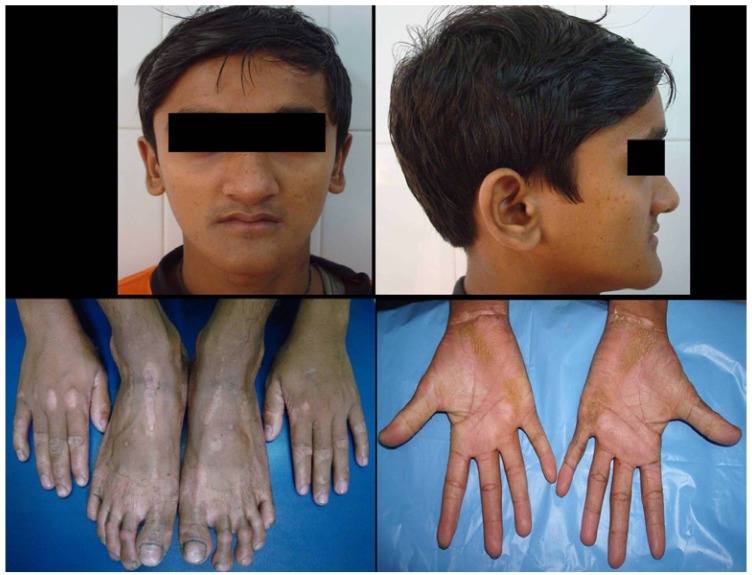
Pre- treatment photographs of the patient showing hyperkeratotic patches on hands and feet.

On clinical examination, the patient presented with a reduced facial height and a senile appearance. There was bilateral palmoplanter keratoderma with symmetric, well-demarcated, yellowish, keratotic plaques on the skin of his palms and soles extending onto the dorsal surfaces (Fig. 1).

**Figure 2:**
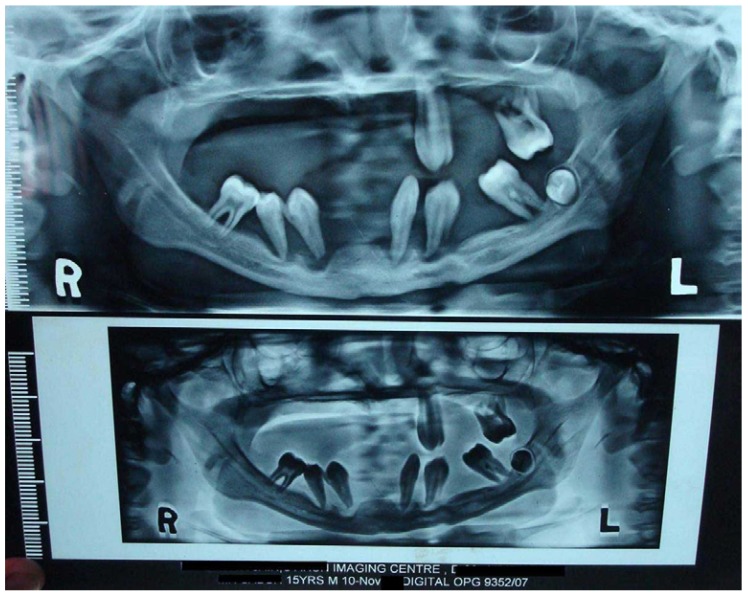
Panoramic radiograph showing “Floating in air” appearance of teeth.

Intraoral examination revealed that most of his teeth were missing and those present were Grade III mobile. The Panoramic radiograph demonstrated advanced bone loss giving the teeth a characteristic “Floating in air” appearance (Fig. 2).

Routine clinical investigations such as complete blood hemogram, liver profile, renal profile and ultrasound of the abdomen and pelvis were all normal. Histopathological examination of gingival tissue as well as punch biopsy of skin of palms and soles revealed mild acanthosis and dense chronic mononuclear infiltrate in the connective tissue.

An assessment of the patient’s intellect using Bhatia’s Battery of Intelligence test revealed that he had an IQ of 97, which falls in the category of Average Intelligence.

Co-relating the characteristic medical and dental history, intraoral findings and laboratory and histopathological investigations, the condition was diagnosed as Papillon-Lefèvre Syndrome. Since all the patient’s remaining teeth had very poor prognosis due to extensive bone loss, they were planned for extraction. Following due informed consent, the extractions were carried out in a phased manner followed by a healing period of 8 weeks, which was uneventful.

**Figure 3:**
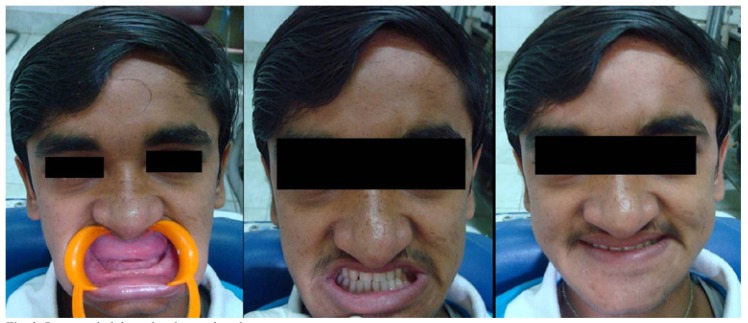
Patient rehabilitated with complete dentures.

Treatment options for prosthodontic rehabilitation were discussed and considering the patient’s age and poor socioeconomic status, a set of complete dentures was planned for now, not ruling out the use of dental implants in future. Complete maxillary and mandibular dentures were delivered (Fig. 3) and the patient was recalled for follow up and necessary adjustments.

The patient was referred to the Department of Dermatology for management of cutaneous lesions where treatment with emollients and Acretenin was initiated.

## Discussion

Papillon-Lefèvre Syndrome was originally described by two French physicians, Papillon and Lefèvre in 1924. The syndrome is inherited as an autosomal recessive trait and has a reported prevalence of 1-4 cases per million (1).

The disorder is characterized by diffuse palmoplantar keratoderma and rapidly progressing periodontitis leading to premature loss of both deciduous and permanent teeth. A third component of dural calcifications has also been reported by Gorlin et al. (2). Almuneef et al. (3) recognize pyogenic liver abscess to be a fairly frequent complication of Papillon-Lefèvre syndrome. The case presented here, however, did not demonstrate any abnormal liver function or ultrasonographic finding.

The dermatological lesions appear first between the ages of 1 to 4 years and include palmoplantar keratosis, varying from mild psoriasiform scaly skin to overt hyperkeratosis. The lesions may be aggravated by cold (4). Our patient gave history of having recurrent pyogenic skin infections since early childhood, a finding which has also been reported by Subramanium et al. (5) and may be attributed to the increased susceptibility to bacterial infections in these patients.

The intraoral presentation in Papillon-Lefèvre Syndrome is characterized by severe periodontitis as early as 3 to 4 years of age. The deciduous teeth develop normally, but their eruption is associated with severe gingival inflammation and subsequent periodontal destruction leading to a premature loss of the primary dentition. A temporary period of healthy gingival tissue is then followed by another phase of destructive periodontitis once the permanent teeth erupt. Affected individuals may thus become partially or completely edentulous in their early teens.

The etiopathogenesis of Papillon-Lefèvre syndrome is relatively obscure. An immunologic basis has been proposed and reduced neutrophil, lymphocyte or monocyte function has been reported in some cases (6). Toomes et al. (7) believe that the syndrome may be genetically determined and have demonstrated loss-of-function mutations affecting both alleles of the lysosomal protease cathepsin-C gene in patients with PLS. The cathepsin-C gene, which is located on chromosome 11q14.1-q14.3 has endopeptidase activity and is expressed in epithelial regions commonly affected by PLS including palms, soles, knees, and keratinized oral gingiva. It is also expressed at high levels in various immune cells including polymorphonuclear leukocytes, macrophages, and their precursors (8,9). Ryu et al. (10) believe that the severe periodontal destruction seen in Papillon-Lefèvre syndrome may be a result of loss of function mutation in the cathepsin C gene and subsequent dysregulation of localized polymorphonuclear leucocytes in inflamed periodontal tissues.

A possible bacterial etiology has also been proposed and it is believed that Actinobacillus actinomycetemcomitans, Porphyromonas gingivalis, Fusobacterium nucleatum and Prevotella intermedia may be amongst the organisms involved not only in periodontal breakdown, but also in the cutaneous lesions of Papillon-Lefèvre syndrome (11).

The management of cases with PLS requires a multidisciplinary approach with the active participation of the dental surgeon, dermatologist and pediatrician. Treatment of the dental component of the disorder is aimed at eliminating the reservoir of causative organisms (12). A treatment protocol for the periodontal therapy in patients with Papillon-Lefèvre syndrome has been proposed by Ullbro et al. (13). Oral retinoids such as acitretin and isotretinoin have proven to be beneficial in treating both the dental and cutaneous lesions of PLS. Retinoid treatment is usually started during the eruption of permanent dentition and is followed till the normal developmental process is complete (14,15).

## Conclusion

Papillon-Lefèvre Syndrome is a devastating disease process, which, due to the associated cutaneous involvement and partial or complete edentulism, can severely affect the psychological, social and esthetic well being of the patient at a very young age. The dental surgeon is often the first to diagnose the syndrome due to the periodontopathia involved. A multidisciplinary approach is imperative, with emphasis not only on the dental and dermatological management, but also on psychological boost up and counseling of the affected individual.
